# Optimization of Phase-Change Material–Elastomer Composite and Integration in Kirigami-Inspired Voxel-Based Actuators

**DOI:** 10.3389/frobt.2021.672934

**Published:** 2021-05-10

**Authors:** Gilles Decroly, Romain Raffoul, Clara Deslypere, Paul Leroy, Louis Van Hove, Alain Delchambre, Pierre Lambert

**Affiliations:** ^1^ TIPs Dpt, Université Libre de Bruxelles, Brussels, Belgium; ^2^ BEAMS Dpt, Université Libre de Bruxelles, Brussels, Belgium

**Keywords:** soft robotics, phase-change material–elastomer composite actuators, active matter, soft material, material optimization, kirigami actuators, voxel-based actuator

## Abstract

Phase-change material–elastomer composite (PCMEC) actuators are composed of a soft elastomer matrix embedding a phase-change fluid, typically ethanol, in microbubbles. When increasing the temperature, the phase change in each bubble induces a macroscopic expansion of the matrix. This class of actuators is promising for soft robotic applications because of their high energy density and actuation strain, and their low cost and easy manufacturing. However, several limitations must be addressed, such as the high actuation temperature and slow actuation speed. Moreover, the lack of a consistent design approach limits the possibility to build PCMEC-based soft robots able to achieve complex tasks. In this work, a new approach to manufacture PCMEC actuators with different fluid–elastomer combinations without altering the quality of the samples is proposed. The influence of the phase-change fluid and the elastomer on free elongation and bending is investigated. We demonstrate that choosing an appropriate fluid increases the actuation strain and speed, and decreases the actuation temperature compared with ethanol, allowing PCMECs to be used in close contact with the human body. Similarly, by using different elastomer materials, the actuator stiffness can be modified, and the experimental results showed that the curvature is roughly proportional to the inverse of Young’s modulus of the pure matrix. To demonstrate the potential of the optimized PCMECs, a kirigami-inspired voxel-based design approach is proposed. PCMEC cubes are molded and reinforced externally by paper. Cuts in the paper induce anisotropy into the structure. Elementary voxels deforming according to the basic kinematics (bending, torsion, elongation, compression and shear) are presented. The combination of these voxels into modular and reconfigurable structures could open new possibilities towards the design of flexible robots able to perform complex tasks.

## 1 Introduction

Active soft matter has gained increased interest for the fabrication of robotic devices with a large range of applications. Safe and flexible soft actuators are proposed as a suitable alternative to solve rigid system limitations, that is, the restricted degrees of freedom and the potential danger for human arising from its rigid interaction with machines (Rus and Michael, 2015). An increasing number of soft materials and transducer are described by Hines et al. (2017), Decroly et al. (2020), targeting application ranging from flexible medical devices to industrial grippers (Cianchetti et al., 2018; Shintake et al., 2018). Among them, pneumatic or fluidic chamber–based actuators, relying on the expansion of a soft structure when submitted to a fluid’s pressure, have been widely studied (Gorissen et al., 2017; Walker et al., 2020). They allow a wide variety of designs and large deformations, but remain limited by the need of a pressure source and pressure leads, and the associated bulkiness, risk of leakage, and rapidly increasing complexity when building complex soft robots (Decroly et al., 2020). Liquid crystal actuators have also been widely studied and present various stimulation modes (Ohm et al., 2010). They are made of crystal molecules that are appended to a compliant polymer backbone. When stimulated thermally or electrically, their molecular reorientation can induce stresses along the backbone that result in a directed actuation strain. Alternatively, dielectric elastomer actuators are composed of an elastomer layer sandwiched between two electrodes, and can undergo shape deformation when subjected to an electric field, and can be used as artificial muscles and for various soft robotic applications (Gupta et al., 2019). Another interesting class of transducer relies directly on the thermal expansion of polymeric materials. Their integration in soft actuators and soft robots has been reviewed by Tian et al. (2020). Various materials with wide ranges of stiffnesses can be used, but their actuation strain remains generally relatively low (Decroly et al., 2020). Finally, swelling or drying transducers rely on the fluid absorption of a matrix (Koetting et al., 2015), and allow the generation of high strains and use with a large range of material stiffnesses and dimensions. However, their chemically triggered actuation remains slow (Decroly et al., 2020).

**FIGURE 1:**
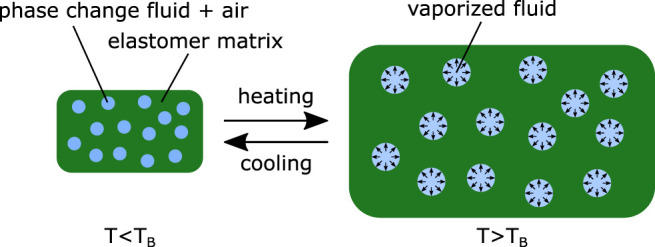
PCMEC actuation principle.

As an alternative, phase-change material–elastomer composite (PCMEC) actuators are made up of a soft elastomer matrix embedding phase-change materials, typically ethanol, in microbubbles (Figure 1). When increasing the temperature, the phase change induces a macroscopic expansion of the matrix. PCMECs have drawn a lot of attention since Miriyev et al. reported the first silicone–ethanol composite in 2017, able to achieve large deformation (up to 900% volumetric strain) and to develop stress up to 1.3 MPa (Miriyev et al., 2017). This class of actuators is promising for a broad range of soft robotic applications because of their energy density and their low cost and easy manufacturing (Miriyev et al., 2017). Moreover, compared to pneumatic actuators, their thermal actuation allows overcoming limitations related to the need of bringing the pressure on the actuation site.

However, several limitations must be addressed to unleash the potential of PCMEC actuators. Their actuation temperature is dependent upon the phase-change material. For ethanol, this corresponds to 78°C, which limits the possibility to use PCMEC actuators in close contact with the human body. Even if the actuation is fully reversible Miriyev et al. (2017), due to the slow diffusion of the fluid out of the composite, its mechanical capabilities degrade with time, and its lifetime remain limited. Up to 120 cycles of actuation have been demonstrated (Xia et al., 2020). Urethane rubber matrix with smaller permeability has been proposed to decrease this diffusion (Noguchi and Tsumori, 2020). Alternatively, it has been shown that PCMECs can be rejuvenated by soaking the samples in ethanol, allowing the fluid to diffuse back into the microbubbles and the PCMEC to regain more than 95% of its actuation capabilities (Miriyev et al., 2018a). Moreover, the actuation and recovery speeds are relatively slow, with typical actuation or recovery times ranging from 10 to 100 s, depending on the thermodynamics of the actuation (Li X. et al., 2020; Noguchi and Tsumori, 2020; Xia et al., 2020). Several actuation methods have been proposed, using hot water or climatic chambers Miriyev et al. (2017), Li X. et al. (2020), Noguchi and Tsumori (2020), embedded heating wires Miriyev et al. (2017), Miriyev et al. (2018b), conductive fabric heaters Cartolano et al. (2019), or the Joule effect allowed by addition of conductive particles in the composite (Bilodeau, 2018; Kapłon et al., 2020). The actuator’s stiffness depends mainly on the elastomer matrix, but its influence remains little studied. PCMECs generally range on the centimeter scale (Li X. et al., 2020). Miniaturization is limited by the bubble size [100 μm to 1 mm; Miriyev et al. (2018b)] and by the diffusion of the phase-change material out of the matrix (Miriyev et al., 2018a). Moreover, the lack of a consistent design approach limits the possibility to build PCMEC-based soft robots able to achieve complex tasks. Only bilayer Miriyev et al. (2018a), Li J. et al. (2020), piston-like (Miriyev et al. (2017), Miriyev et al. (2018b), and McKibben-like Miriyev et al. (2017), Bilodeau (2018) configurations have been demonstrated so far.

This works aimed at proposing strategies to overcome some of these limitations. A new approach to manufacture samples with different fluid–elastomer combinations without altering the quality of the samples is proposed, allowing to modulate the actuation temperature of the PCMEC. Two experimental studies investigated the influence of the phase-change fluid and the elastomer on the mechanical capabilities of unconstrained and bilayer PCMEC structures. Actuation strain and the strain rate are used to characterize the influence of the fluid on free elongation, and curvature is used to characterize bending on simple structures made of different elastomers. Other intrinsic limitations of PCMECs such as the complex miniaturization, the need of frequent rejuvenation, and the relatively slow actuation time are not addressed in this work. Going further toward the functionalization of PCMEC actuators, elementary voxels capable of elongation, compression, shear, bending, and twisting are designed and implemented using kirigami-inspired paper reinforcement. This voxel-based approach could be the first step to design modular soft robots able to achieve complex kinematics.

## 2 Influence of the Phase-Change Fluid

To overcome the previously described PCMEC limitations, the influence of the phase-change fluid is first investigated. Replacing ethanol by a fluid having adequate thermodynamic properties, such as a lower boiling point or latent heat of evaporation, could allow us to customize the actuation temperature, and increase its actuation strain, used here to characterize the PCMEC mechanical properties.

### 2.1 Materials and Method

In previous work, Noguchi and Tsumori fabricated PCMECs with water and surfactant, and with fluorine-based fluid Novec (Sigma-Aldrich Novec™ 7,000 Engineered Fluid), with boiling points of, respectively, 100 and 34°C (Noguchi and Tsumori, 2020). Alternatively, other studies used the vaporization of a fluid—this time inside a soft chamber as an actuation method. As PCMEC actuators, these designs allow remote actuation to avoid the need of a pressure source, but remain subject to leaks. Ethanol Han et al. (2019), Chellattoan et al. (2020), Kang et al. (2070), water Li J. et al. (2020), FC-72 (3M™ Fluorinert™ Electronic Liquid FC-72) Matsuoka et al. (2016), acetone Chellattoan et al. (2020), or Novec (Boyvat et al., 2019; Narumi et al., 2020; Ueno and Monnai, 2020) was reported.

For this study, several fluids have been identified as alternatives for ethanol and are presented in Table 1. The toxicity of the gas was taken as a primary factor to identify the potential fluids, since PCMEC actuators are intended to be used in close interaction with humans. Based on the Occupational Safety and Health Administration (OSHA) recommendation, only fluids with a toxicity threshold of 40 ppm or more were considered. Novec (Sigma-Aldrich Novec™ 7, 000 Engineered Fluid) and FC-72 (3M™ Fluorinert™ Electronic Liquid FC-72) present low boiling points and have been reported in the literature as phase-change fluid for soft robotic applications. Additionally, ethanol, acetone, and isopropanol are usual lab chemicals and were included in the study. Two fluids were added to broaden the parameter space: methanol for its particularly high latent heat of vaporization and acetonitrile for its high boiling point. Their properties are presented in Table 1. The data were collected from the fluid datasheets and completed from PubChem (https://pubchem.ncbi.nlm.nih.gov/), Chemeo (https://www.chemeo.com/), and the OSHA database (https://osha.europa.eu/fr) for toxicity-related data.

**TABLE 1:** Selected fluid properties. The density and molar concentration are given at 1 atm and 20°C.

	Boiling point	Latent heat	Density	Molar mass	Molar concentration	Toxicity threshold
	TB (°C)	∆Hvap (kJ/mol)	*ρ* (g/L)	M (g/mol)	c (mol/L)	(ppm)
Novec	34	28.4	1400	200	7	250
FC72	56	29.7	1680	338	5	
Acetone	56.1	29.1	784	58.0	13.5	1000
Methanol	64.5	35.2	792	32.0	24.8	200
Ethanol	78.2	38.6	789	46.1	17.1	1000
Isopropanol	80.3	39.9	786	60.1	13.1	400
Acetonitrile	81.6	33.2	786	41.1	19.1	40

This study focuses on the boiling point, the latent heat of vaporization, and the molar mass of the fluids. The importance of the boiling point *T*_*B*_ is straightforward, allowing to decrease the actuation temperature. The latent heat of vaporization ∆*H*_*vap*_ [kJ/mol] is expected to play a role in both the actuation dynamics and the actuation strain, since it reflects the energy the fluid requires to vaporize xand is linked to the saturation pressure *P*_*sat*_ by the Clausius–Clapeyron relation, when considering ∆*H*_*vap*_ as constant: $P_{sat}=P_{0}.e^{\frac{\text{Δ}H_{vap}}{R}\left(\frac{1}{T}-\frac{1}{T_{0}}\right)}$, where *P*_0_ is the reference pressure at the reference temperature *T*_0_, *R* the perfect gas constant, and *T* the temperature. Note that the necessary assumptions, that is, low pressure and low temperature compared to the critical point, are not verified here, with the aim being only to identify key parameters for our experimental study. Li et al. developed a micromechanical model for ethanol-based PCMECs and showed that for small temperature variations, the coefficient of thermal expansion (CTE) of the PCMEC can be expressed as a function of the fluid volume fraction, the elastic properties of the silicone matrix, and the variation of pressure in the microbubbles due to fluid vaporization (Li X. et al., 2020). Additionally, it can be expected that at a given temperature, the expansion of the material will depend on the number of moles of the fluid contained in the microbubbles. This can be characterized by the molar concentration *c* at 1 atm and 20°C that can be computed from the molar mass *M* and the density ρ $\left(c=\frac{\rho }{M}\right)$. Note that the impact on the environment is an important factor to take into account and that life-cycle assessment should be conducted for each fluid, considering future use of PCMEC actuators in soft robotics.

**FIGURE 2:**
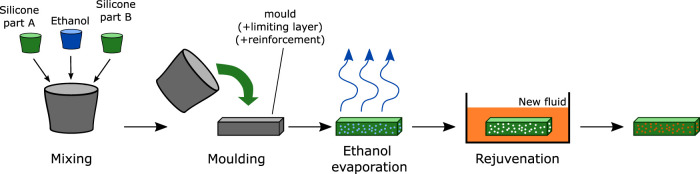
Fabrication process of the PCMEC actuators.

Ethanol-based PCMECs are classically manufactured by adding ethanol when mixing the two-part silicone, allowing the microbubbles to form during the curing (Miriyev et al., 2017). In previous work, Miriyev et al. studied the influence of the ethanol fraction and found that a composition of 20% in volume was optimal for applications in robotics (Miriyev et al., 2018b). Noguchi and Tsumori proposed to replace ethanol during the fabrication process, even by water with surfactant, or by Novec (Noguchi and Tsumori, 2020). After mixing in a stirring deaerator and with an additional step of microwave heating to activate the samples, it allowed to fabricate PCMECs with smaller microbubbles because of the fluid’s respective low surface tension and high miscibility in the silicone matrix. However, this process remains limited to these specific fluids (water with surfactant or Novec) and is not suitable as is for any fluid. Moreover, in preliminary work, we obtained poor results when manually directly mixing the phase-change fluid with the two-part silicone, depending on the fluid (imperfect curing, microbubbles of variable size and circularity, or no bubbles at all). To overcome this limitation, we propose to adapt the rejuvenation process proposed by Miriyev et al. (2018a) to refill the microbubbles with the desired fluid, as presented in Figure 2. The sample is prepared by mixing manually the ethanol and the two-part uncured silicone for several minutes and pouring the mix into a mold. After curing (several hours, depending on the silicone), the sample is unmolded and is left at room temperature for at least two days, to allow the ethanol to diffuse outside of the microbubbles. Note that the duration of this phase depends on the dimensions of the samples. As the last step, the sample is soaked in the new fluid for an equivalent time, allowing it to fill the microbubbles by diffusion. This process allows to fabricate reproducible high-quality samples, regardless of the phase-change fluid, if it can diffuse into the silicone matrix, increasing strongly the process duration but not its complexity.

**FIGURE 3:**
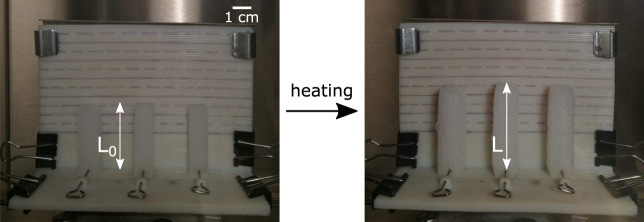
Experimental actuation strain measurement setup.

To study the influence of the fluid, unconstrained samples of 10 mm × 10 mm × 40 mm are prepared. Ecoflex 00–50 (Smooth-On, Inc.) is used as an elastomer material. Its properties are detailed in Table 2. The samples are prepared in vertical 3D printed molds, and the top is cut to remove the imperfections due to microbubble accumulations at the surface of the sample. The experimental setup for the actuation strain measurement is shown in Figure 3. The samples are placed vertically on a support and anchored by a needle at the bottom, in a temperature-controlled climatic chamber. Their expansion is measured, allowing a straightforward characterization of the actuation capabilities of the PCMEC. The sample is considered to expand isotropically so that only the vertical expansion of the sample is measured. The actuation strain is defined as $\epsilon =\frac{l-l_{0}}{l_{0}}$, with the initial length and *l*_0_ the actuated length. Using this normalized quantity allows to characterize the PCMEC independently of the geometry of the structure and to compare the results with the literature.

**TABLE 2:** Silicone properties. Values from the datasheets.

	Shore hardness	100% modulus *E* (MPa)	Uncured viscosity (Pa.s)	Elongation at break (%)	Tensile strength (MPa)
Ecoflex 00–30 (E30)	00–30	0.069	3	900	1.38
Ecoflex 00–50 (E50)	00–50	0.083	8	980	2.17
Dragon skin 10 (D10)	10A	0.152	20	1,000	3.28
Dragon skin 20 (D20)	20A	0.338	20	620	3.79

### 2.2 Results

**FIGURE 4:**
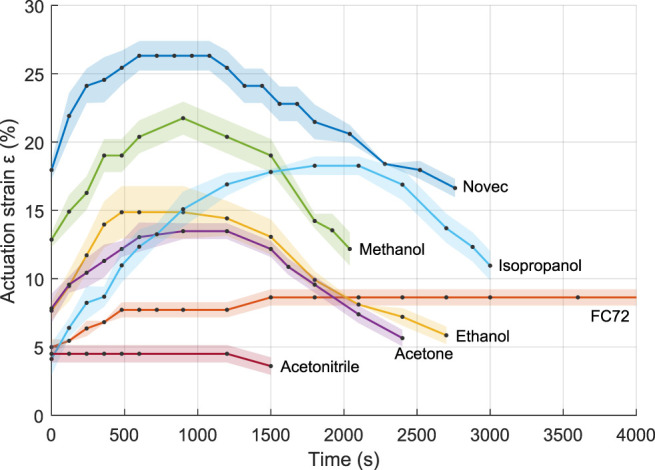
Actuation strain as a function of the time for the different fluids, response to a 5° temperature step above boiling. The shaded areas represent the standard deviation; the data points are in black.

As a first approach, the response of the different fluid–elastomer combinations to a 5°C step above the boiling point is investigated. The samples are placed in the climatic chamber, which was heated at the boiling point of the fluid. After thermalization of the samples, the temperature of the chamber is increased by 5°C. For each fluid, the expansion of three sample is measured simultaneously. The results are shown in Figure 4. At the boiling point of each fluid, an expansion of the sample is already observed, explaining the initial strain. Note that given the relatively low temperature (only 5°C above the boiling point), the achieved strain remains way lower than the maximal achievable strains. The results are presented in Figure 4. Three “phases” are observed: a first phase when the actuation strain increase is maximal, a second phase of stabilization, and a leakage phase, where the actuation strain decrease because of the diffusion of the fluid outside of the material. It can be observed from Figure 4 that, as expected, the different fluids influence the actuation properties. Novec-based samples reach higher strain than other fluids, while no significant expansion is observed for acetonitrile. The strain rate also varies between the samples as well as the time to reach the maximal actuation. Finally, the leakage rate is also strongly dependent on the fluid. Note that for FC-72, this leakage was only observed after approximately 8000 s. This slow leakage rate indicates a low diffusivity of the fluid in the matrix, suggesting that the rejuvenation of the samples could be incomplete and that higher actuation strains could be achieved by allowing the samples to be soaked longer in the fluid.

**FIGURE 5:**
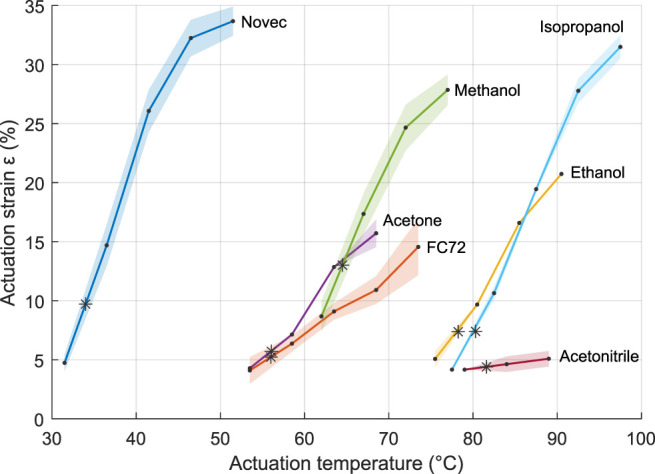
Actuation strain as a function of the temperature for the different fluids. The shaded areas represent the standard deviation; the data points are in black. The stars indicate the boiling points.

Additionally, the influence of the temperature on the actuation strain is further studied. For each fluid, the temperature is increased 5 by 5°C, starting at 2.5°C below the boiling point. The duration of each step is set to 75% of the total raise time obtained in the first test. Given the slow actuation, leakage occurs, and the achieved strain remains relatively low. It can be expected that higher strain values can be obtained with adequate setups. This is further investigated in the following paragraph. The results are presented in Figure 5. The actuation occurs in a temperature range of 15–30°C, mainly above the boiling point, and the actuation temperature can be strongly decreased compared to ethanol-based samples. Moreover, the slope of the strain–temperature curves gives information of the controllability of the different fluid-based PCMECs. A steep slope indicates a smaller temperature range of actuation, and less control in a given strain value must be reached. Again, Novec, ethanol, methanol, and isopropanol-based samples show the largest expansion.

**FIGURE 6:**
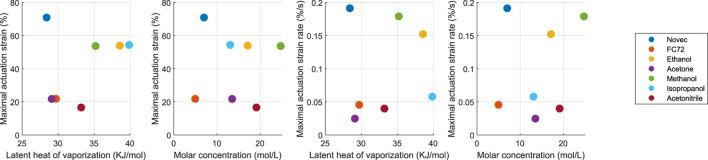
Influence of the fluid parameters on the maximal strain *ϵ* and a maximal strain rate $\dot{\epsilon }$. Experimental results.

Finally, to evaluate the maximal actuation strain and the strain rate, the response of the samples when subjected to a temperature 30°C above its boiling point is investigated. For this test, the samples are placed in the preheated chamber directly from room temperature. Each measure is repeated three times. The actuation strain is measured after 5 min $\left(\epsilon_{5}\right)$ and when the maximum is reached $\left(\epsilon_{max}\right)$. The maximal strain rate is defined as ${\dot{\epsilon }}_{max}=\epsilon_{5}/300\;s$. From this, the strain rate can be calculated with actuation time to reach a strain of 10%, defined here as $t_{10\text{\%}}=10/{\dot{\epsilon }}_{max}$. The results are presented in Table 3; Figure 6. As in the previous tests, Novec and alcohol-based PCMECs show the largest actuation strain and the strain rate, with an exception made from isopropanol, having a relatively slow strain rate. In terms of absolute strain values, extremely high strain is obtained, and Novec-based samples exceed previously reported ethanol-based sample with approximately 30%. Alternatively, methanol-based samples reach similar actuation strains, but with a higher strain rate than ethanol. The maximal strain and strain results are represented as a function of the latent heat and the molar concentration in Figure 6. No clear trend appears from the results, suggesting that the latent heat of vaporization and the molar concentration are not sufficient to capture the influence of the fluid on the mechanical capabilities. Even if a previously developed micromechanical model allowed a satisfying understanding of the phenomena Li X. et al. (2020), they remain limited to ethanol and to small temperature variation ranges, and are not sufficient to model the influence of the different fluids. Complete thermodynamic models should be developed for a better understanding of the actuation.

**TABLE 3:** Maximal actuation strain, the strain rate, and actuation time for the different fluids, response to a temperature step from room temperature to 30°C above the boiling point.

	Maximal actuation strain ϵ_max_ (%)	Maximal strain rate $\dot{\epsilon }$_max_ (%/s)	10% expansion time *t*_10_% (s)
Novec	70.9 ± 2.3	0.191 ± 0.007	52.3 ± 1.9
FC72	21.8 ± 1.1	0.046 ± 0.004	221.7 ± 20.5
Acetone	21.7 ± 0.7	0.025 ± 0.002	408.0 ± 33.9
Methanol	53.4 ± 2.8	0.179 ± 0.009	56.0 ± 2.7
Ethanol	53.9 ± 3.2	0.152 ± 0.011	66.0 ± 4.5
Isopropanol	54.4 ± 0.9	0.058 ± 0.002	173.1 ± 5.0
Acetonitrile	16.6 ± 1.2	0.040 ± 0.002	251.1 ± 13.4

## 3 Influence of the Elastomer Material

The elastomer matrix, and in particular its stiffness, is also expected to have an influence on the mechanical capabilities of PCMEC actuators. In this work, four silicones with different stiffnesses are investigated. Simple bilayer structures are built, and their curvature is measured to characterize the actuation capabilities.

### 3.1 Materials and Methods

Most of the previously reported PCMECs used Ecoflex silicones (Ecoflex series, Smooth-On, Inc.), a series of two-part platinum-catalyzed silicone. Ecoflex 00–50 (E50) Miriyev et al. (2017), Ecoflex 00–35 (Li X. et al. (2020), and Ecoflex 00–10 are reported. Alternatively, the use of urethane rubber, with a lower gas permeability, was also demonstrated but it showed less efficiency in preserving the samples shaped upon actuation (Noguchi and Tsumori, 2020). In this work, we selected four platinum-catalyzed silicones for the Ecoflex and Dragon Skin series (Smooth-On, Inc.). Their properties, extracted from the datasheet, are presented in Table 2. Silicones are known to be hyperelastic materials, that is, characterized by large nonlinear elastic deformations (Marechal et al., 2019). Li X. et al. (2020) proposed a model to estimate the Young’s modulus of ethanol-based PCMECs, based on elastomer properties and on the volume fraction of ethanol (Li X. et al., 2020). Here, a simpler approach is preferred, and a Young’s modulus value is approximated by the 100% modulus of the silicones (Table 2).

**FIGURE 7:**
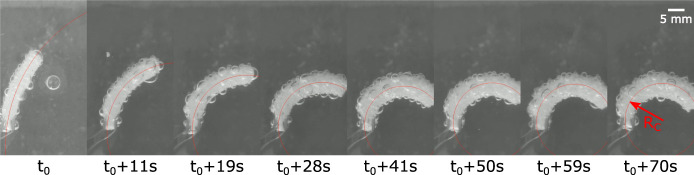
Influence of the elastomer materials on the bending of bilayer structures. The inextensible paper layer is on the right side of the sample. The best-fitting circle is in red.

Going one step further toward the functionalization of the PCMEC, bilayer bending structures are developed. Using the fabrication process described in Figure 2, 5 mm × 5 mm × 30 mm samples are prepared. An inextensible paper layer of 5 mm × 30 mm is added in the bottom of the mold before pouring the uncured silicone–ethanol mix. During actuation, this inextensible layer constrains the expansion of the PCMEC, acting as a neutral fiber, and the sample bends toward it. Only ethanol and Novec that showed the best results were used as phase-change fluid, in combinations with the different silicones. Each sample is placed from room temperature into a temperature-controlled water bath, maintained at a temperature of 10°C superior to the boiling point of the fluid. Each test is repeated three times. Using video capture, the skeleton of the sample is extracted, and the best-fitting circle is computed. The setup is shown in Figure 7, with the best-fitting circle represented in red.

### 3.2 Results

**FIGURE 8:**
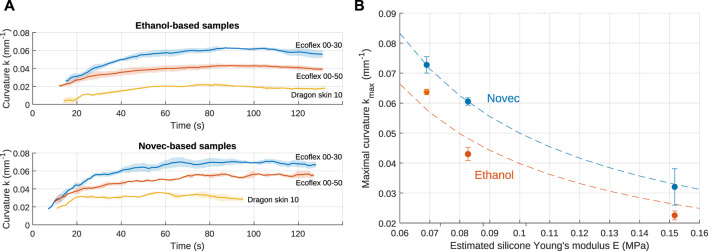
Influence of the elastomer on the bending capabilities when heated at 10 °C above the fluid boiling points. **(A)** Raw results for ethanol and Novec for different silicones. The shaded areas represent the standard deviation. **(B)** Maximal curvature for each fluid–material combination. The dashed lines are fitted curves $k∼\epsilon =\frac{1}{E}$.

The curvature, defined as $k=\frac{1}{R_{c}}=\frac{\theta }{L}$, with *R*_*c*_ as the radius of curvature, that is, the radius of the best-fitting circle, θ the bending angle, and *L* the length of the sample, is used to characterize the bending, since it does not depend on the sample length *L* (Decroly et al., 2020). Furthermore, under the assumption of constant curvature, the actuation strain is directly proportional to the curvature and can be obtained from the most elongated fiber (at a distance *d* from the neutral axis) of the actuator: ϵ=kd (Decroly et al., 2020). The bending results are shown in Figure 8A. As expected, larger curvature is obtained with the softest materials and with Novec-based samples. Since the samples are smaller than those of the free-elongation characterization, maximal actuation is reached faster. An effect of the leakage can already be observed after ∼100 s. Because of the high matrix stiffness, no significant bending was obtained with D20. The maximal curvature obtained for each sample is shown in Figure 8B, where the curvature is represented as a function of the estimated Young’s modulus *E*. For linear elastic materials, the stress and strain are related by the relation $\epsilon =\frac{\sigma }{E}$. As a first approach, it can be understood that the actuation stress generated by the microbubble expansion will result in a strain, and consequently a curvature, inversely proportional to the matrix stiffness: $k∼\epsilon ∼\frac{1}{E}$. Such best-fitting curves are represented on the figure, and the experimental results follow roughly the expected dependency.

## 4 Toward PCMEC Functionalization: A Voxel-Based Approach

Several functional structures have been build using PCMECs, from simple bilayer Miriyev et al. (2018a), Li J. et al. (2020) to piston-like Miriyev et al. (2017), Miriyev et al. (2018b) and McKibben-like Miriyev et al. (2017), Bilodeau (2018) artificial muscles. Other original designs have been demonstrated to build PCMEC actuators capable of locomotion (Li X. et al., 2020; Noguchi and Tsumori, 2020). However, the lack of a consistent design methodology limits the possibility to build PCMEC-based soft robots able to achieve complex tasks, such as gripping, manipulation, locomotion, or configuration change (Decroly et al., 2020). To produce a motion along a given degree of freedom, the PCMEC expansion must be guided in the desired way. PCMECs expand isotropically and require consequently the addition of mechanical reinforcement to the materials. Such methods for generating various kinematics have been proven able to achieve a wide range of motions but were mostly implemented on pneumatic or fluidic chamber actuators (Gorissen et al., 2017). Several methodologies and models have been developed to program the motion of the actuators, typically using external fiber of fabric reinforcement (Decroly et al., 2020). If considering a beam with a fixed extremity, its free end has the six degrees of freedom: two in bending, two in shear, one in twisting, and one in elongation/compression. Bending, twisting, expansion, and contraction (McKibben-like actuators) can easily be obtained with external reinforcement Connolly et al. (2017), Decroly et al. (2020), but, to our knowledge, shear has never been demonstrated. Recently, Jin et al. demonstrated the possibility to customize finely and locally the deformation of pneumatic structures, using kirigami-inspired reinforcement, made of patterned cut in an inextensible membrane (Jin et al., 2020). Using a similar principle with PCMECs would allow us to overcome the need of pressure source and pressure lines, and the associated leakages and bulkiness limitations. In this work, as a first step, we develop elementary voxels which are able to cover the six elementary degrees of freedom (translations and rotations).

### 4.1 Materials and Methods

**FIGURE 9:**
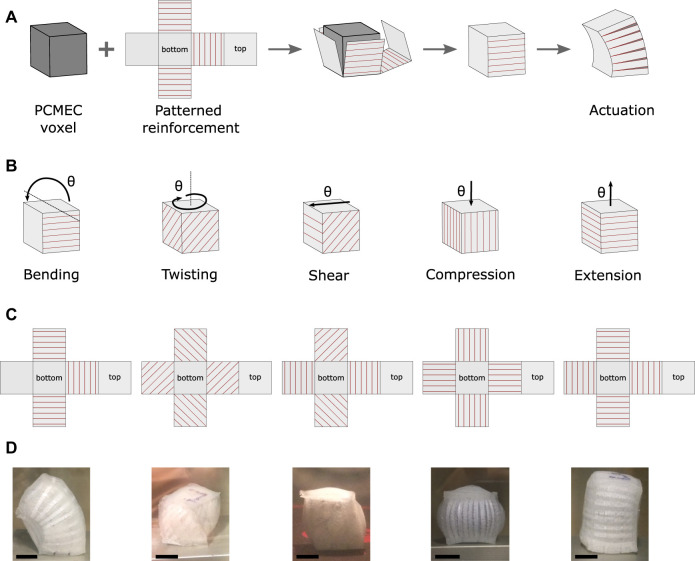
PCMEC voxels. **(A)** Principle illustrated for bending. The cuts in the paper reinforcement are indicated in red. **(B)** Elementary kinematics, pure deformations. **(C)** Kirigami patterns. **(D)** Experimental results.

The working principle of the voxels is presented in Figure 9A. 20 mm × 20 mm × 20 mm PCMEC cubes, called here as voxels, are molded and reinforced externally by kirigami-inspired paper. The cuts in the paper induce the anisotropy into the structure. Five elementary voxels deforming according to basic kinematics are made: bending, twisting, elongation/compression, or shear (Figure 9B). For each kinematic, a specific pattern is designed. The fabrication process presented in Figure 2 is used. The paper reinforcement is added in the mold before pouring the uncured mix, and a 3-mm layer of silicone is added externally afterward, to ensure good adhesion of the paper. The implemented designs are made of Ecoflex 00–30 (E30) and ethanol. A climatic chamber is used for actuation. The voxels are heated at 10°C above the boiling point of ethanol, to achieve a maximal actuation while preventing the paper reinforcement from tearing.

### 4.2 Results

The designed kirigami-inspired reinforcements and the experimental results are shown in Figures 9C,D. All the kinematics can be obtained with adequate reinforcement. To our knowledge, this is the first time that shear is reported. It is interesting to note that for most of the kinematics, “pure” deformation cannot be obtained. To obtain a deformation θ along the desired degree of freedom from a variation of volume *V* of the voxel, the following condition must be verified: $\frac{\partial V}{\partial \theta }>0$. It can be understood that the deformation along θ must allow an increase of volume of the voxel. Consequently, only pure elongation can be obtained. Pure bending, twisting, and shear (illustrated in Figure 9B) would result in zero volume variation. Hence, the reinforcement must allow a volume increase in the form of unwanted elongation. Similarly, for compression, unwanted radial expansion compensates the volume change.

## 5 Discussion

**FIGURE 10:**
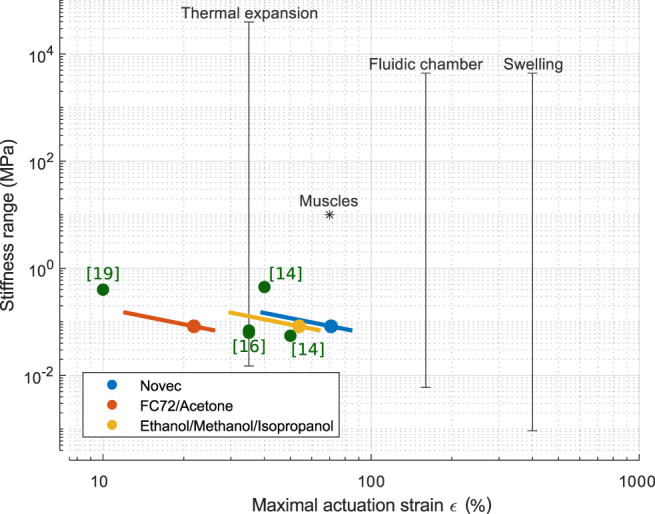
Comparison of the experimental results with the literature in a stiffness–strain diagram. Previously reported PCMEC actuators are represented in green. The black lines represent other relevant classes of transducers and indicate the maximal strain and the extreme stiffness values reported in the literature (Decroly et al., 2020). The data points are completed by lines ∼1/ϵ covering the investigated stiffness range.

In this work, we demonstrated that choosing an appropriate phase-change fluid increases the actuation strain and speed, while decreasing the actuation temperature compared with ethanol. We also demonstrated the possibility to modify the stiffness of the PCMEC and showed that experimentally, the maximal strain will be inversely proportional to the matrix stiffness. Figure 10 compares the experimental results with the literature in a strain–stiffness diagram. Previously reported PCMEC actuators are represented, as well as transducers that could be used in similar designs as those presented in this work, that is, transducers showing isotropic expansion. For muscles and other transducers (fluidic chamber, thermal expansion, and swelling), the stiffness range and maximal reported strain are collected from (Decroly et al., 2020). The experimental results’ data points are completed by lines ∼1/ϵ, covering the investigate stiffness range and following the experimental modeling of the stiffness influence. The ethanol-based actuators show coherent results with previously reported PCMECs, while replacing ethanol by Novec allows to reach similar strain than muscles. Compared to other transducers, PCMECs remain relatively soft, but allow larger actuation strain than thermal expansion while reaching similar values as typical fluidic chamber actuators (the abscissa of the lines represents the maximal value reported in the literature). Concerning the actuation time, even if the actuation remains relatively slow (10% strain reached between 50 and 400 s depending of the fluid), we showed that this can be modulated with an appropriate choice of phase-change fluid. Moreover, the actuation time depends mainly on the thermodynamics of the actuation and on the actuator’s dimensions, and previous work demonstrated that an adequate actuation method can reduce the actuation time.

**FIGURE 11:**
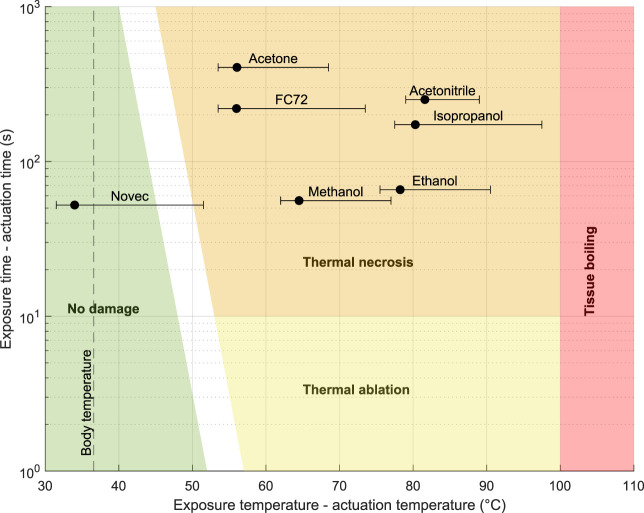
Experimental results placed in an actuation temperature–actuation time diagram and superposed to the effects of the temperature on the tissues as a function of the exposure time. The actuation time is estimated to reach an actuation strain of 10%. The fluid boiling point is indicated by the dots and the range is extracted from Figure 5.

Toward the design of PCMEC-based soft devices, Novec showed to be an excellent candidate, with a boiling point as low as 34°C. This allows to overcome one of the main limitations of PCMEC actuators and to increase the potential of such devices to be used in contact with human bodies, for example, in biomedical devices. Figure 11 represents the effect of temperature on human tissues as a function of the exposure time: he longer the exposure time, the lower the safe limit for temperature. Each studied fluid-based PCMEC is represented in this diagram. The actuation time is estimated to reach an actuation strain of 10%, and the temperature ranges are extracted from Figure 5. They correspond to the range of temperatures around the boiling points for which expansion is observed. Using Novec allows us to reach the “no-damage” zone of the diagram, meaning that actuation can be used in contact with the human body without time limitation or thermal insulation.

Moreover, this voxel-based approach aims to be a first step toward a modular design methodology for soft robots. Compared with previous work using fluidic actuation and anisotropic reinforcement, we demonstrated that shear can also be obtained and that this design principle can be generalized to any isotropic expansive transducers. Combining these voxels into modular structures could open new possibilities toward the generation of complex motions, that is, deformations beyond pure elongation, shear, twist, or bending. In previous work, reconfigurable robots capable of locomotion have been built by assembling pneumatically actuated voxels able only of contraction and expansion Zou et al. (2018), Sui et al. (2019). Lin et al. designed more complex and versatile soft robots, using bending and contractile pneumatic voxels Lin et al. (2020), but requiring an important amount of pressure lines and complex pressure signals. The heat-actuated fixed kinematic voxels presented in this work compromise among the design simplicity, ease of actuation, and the loss of reconfigurability of the voxels. Next steps in the development of the methodology will focus on questions related to the assembly and actuation of the voxels to achieve a given kinematics.

## 6 Conclusion

A new approach to manufacture PCMEC actuators with different fluid–elastomer combinations without altering the quality of the samples has been validated. We demonstrated that choosing an appropriate fluid allows us to increase the actuation strain and speed and to decrease the actuation temperature compared with ethanol. In particular, using fluorine-based fluid Novec allows us to decrease the actuation temperature to 34°C and to increase the maximal actuation strain by 30% compared to ethanol-based samples. However, the influence of the fluid’s properties remains mostly not understood. Similarly, by using different elastomer materials, the actuator stiffness can be modified, and the experimental results showed that the curvature is roughly proportional to the inverse of Young’s modulus of the pure matrix. To demonstrate the potential of the optimized PCMEC, a kirigami-inspired voxel-based design approach has been proposed. As a proof of concept, elementary voxels deforming according to the basic kinematics are presented: bending, twisting, elongation, compression, and shear. To our knowledge, this is the first time that a shear actuator is reported in the literature. Combining these voxels into modular and reconfigurable structures could open new possibilities toward the design of soft robots able to perform complex tasks.

Future work should focus on the development of thermo- and micromechanical models, to allow a deeper understanding of the influence of the fluid’s properties on the actuation and draw guidelines for fluid selection for given requirements in terms of actuation temperature and mechanical output. Moreover, the first step toward a voxel-based approach for designing soft robots is presented in this work. As next steps, an adequate actuation method should be developed, and design rules for assembling the voxels to achieve a given complex kinematics should be further investigated. The influence of such PCMEC-based soft robots on the environment should also be further investigated through life-cycle assessment.

## Data Availability

The raw data supporting the conclusions of this article will be made available by the authors, without undue reservation.
